# Mp1p Is a Virulence Factor in *Talaromyces (Penicillium) marneffei*

**DOI:** 10.1371/journal.pntd.0004907

**Published:** 2016-08-25

**Authors:** Patrick C. Y. Woo, Susanna K. P. Lau, Candy C. Y. Lau, Edward T. K. Tung, Ken T. K. Chong, Fengjuan Yang, Hongmin Zhang, Raymond K. C. Lo, Jian-Pao Cai, Rex K. H. Au-Yeung, Wing-Fung Ng, Herman Tse, Samson S. Y. Wong, Simin Xu, Wai Hei Lam, Man-Kit Tse, Kong Hung Sze, Richard Y. Kao, Neil E. Reiner, Quan Hao, Kwok-Yung Yuen

**Affiliations:** 1 Department of Microbiology, The University of Hong Kong, Hong Kong; 2 State Key Laboratory of Emerging Infectious Diseases, The University of Hong Kong, Hong Kong; 3 Research Centre of Infection and Immunology, The University of Hong Kong, Hong Kong; 4 Carol Yu Centre for Infection, The University of Hong Kong, Hong Kong; 5 School of Biomedical Sciences, The University of Hong Kong, Hong Kong; 6 Department of Pathology, The University of Hong Kong, Hong Kong; 7 Department of Pathology, United Christian Hospital and Tseung Kwan O Hospital, Hong Kong; 8 Department of Medicine, University of British Columbia, Vancouver, Canada; University of California San Diego School of Medicine, UNITED STATES

## Abstract

**Background:**

*Talaromyces marneffei* is an opportunistic dimorphic fungus prevalent in Southeast Asia. We previously demonstrated that Mp1p is an immunogenic surface and secretory mannoprotein of *T*. *marneffei*. Since Mp1p is a surface protein that can generate protective immunity, we hypothesized that Mp1p and/or its homologs are virulence factors.

**Methodology/Principal Findings:**

We examined the pathogenic roles of Mp1p and its homologs in a mouse model. All mice died 21 and 30 days after challenge with wild-type *T*. *marneffei* PM1 and *MP1* complemented mutant respectively. None of the mice died 60 days after challenge with *MP1* knockout mutant (P<0.0001). Seventy percent of mice died 60 days after challenge with *MP1* knockdown mutant (P<0.0001). All mice died after challenge with *MPLP1* to *MPLP13* knockdown mutants, suggesting that only Mp1p plays a significant role in virulence. The mean fungal loads of PM1 and *MP1* complemented mutant in the liver, lung, kidney and spleen were significantly higher than those of the *MP1* knockout mutant. Similarly, the mean load of PM1 in the liver, lung and spleen were significantly higher than that of the *MP1* knockdown mutant. Histopathological studies showed an abundance of yeast in the kidney, spleen, liver and lung with more marked hepatic and splenic necrosis in mice challenged with PM1 compared to *MP1* knockout and *MP1* knockdown mutants. Likewise, a higher abundance of yeast was observed in the liver and spleen of mice challenged with *MP1* complemented mutant compared to *MP1* knockout mutant. PM1 and *MP1* complemented mutant survived significantly better than *MP1* knockout mutant in macrophages at 48 hours (P<0.01) post-infection. The mean fungal counts of *Pichia pastoris* GS115-*MP1* in the liver (P<0.001) and spleen (P<0.05) of mice were significantly higher than those of GS115 at 24 hours post-challenge.

**Conclusions/Significance:**

Mp1p is a key virulence factor of *T*. *marneffei*. Mp1p mediates virulence by improving the survival of *T*. *marneffei* in macrophages.

## Author Summary

*Talaromyces* (*Penicillium*) *marneffei* is an opportunistic thermal dimorphic fungus most prevalent in Southeast Asia. Our team has previously shown that Mp1p, a protein encoded by the *MP1* gene, is an immunogenic surface and secretory protein of *T*. *marneffei*. In this study, we showed that mice challenged with *T*. *marneffei* with the *MP1* gene died but those challenged with *T*. *marneffei* without the *MP1* gene did not die. There was also significantly higher fungal load and more necrosis in organs of mice challenged with *T*. *marneffei* with the *MP1* gene than *T*. *marneffei* without the *MP1* gene. Furthermore, *T*. *marneffei* with the *MP1* gene survived better in macrophages than *T*. *marneffei* without the *MP1* gene and *Pichia pastoris* with the *MP1* gene survived in mice better than *P*. *pastoris* without the *MP1* gene. Our data support that Mp1p is a key virulence factor of *T*. *marneffei* and Mp1p mediates virulence by improving the survival of *T*. *marneffei* in macrophages.

## Introduction

*Talaromyces* (*Penicillium*) *marneffei* is an opportunistic thermal dimorphic fungus most prevalent in Southeast Asia [1–6]. Bamboo rats and the soil of their burrows are known to be important sources of *T*. *marneffei*. Since the 1980s, a marked increase in the number of infections caused by *T*. *marneffei* has been observed, primarily as a result of the HIV pandemic. In addition to tuberculosis and cryptococcosis, *T*. *marneffei* infection is one the most important indicator of AIDS in our locality. However, in recent years, there has been a surge in the number of *T*. *marneffei* infections in HIV-negative patients owing to the use of a variety of immunosuppressive therapies and also due to the increased recognition of underlying immunodeficiency syndromes [3, 7–11].

The first line defense of the human body against *T*. *marneffei* infection is achieved mainly through tissue macrophages; however, the mechanisms by which *T*. *marneffei* evades host defense is not well understood [12, 13]. In 1998, we described the cloning of a cell surface and abundantly secreted immunogenic mannoprotein, Mp1p, in *T*. *marneffei* [14]. Mp1p is a 462-amino acid protein with two homologous domains, which are named as lipid binding domain 1 (Mp1p-LBD1) and lipid binding domain 2 (Mp1p-LBD2). We demonstrated that Mp1p based enzyme-linked immunosorbent assays (ELISAs) can be used for antigen and antibody detection in patients with *T*. *marneffei* infections and showed that Mp1p has the ability to generate protective immunity in mice [15–17]. Through analysis of the genome sequence of *T*. *marneffei*, we observed the presence of 13 Mp1p homologs in its genome [18]. Moreover, the amino acid sequence of Mp1p in different strains of *T*. *marneffei* was found to be highly variable, especially in Mp1p-LBD1; and by using Mp1p and four additional Mp1p homologs, we constructed a multilocus sequence typing scheme for *T*. *marneffei* [19]. Recently, we have solved the X-ray crystallographic structure of Mp1p-LBD2, the relatively more conserved LBD of Mp1p, and have shown that it is able to bind palmitic acid [20].

Since Mp1p is a surface protein that can generate protective immunity, we hypothesize that Mp1p and/or its homologs are virulence factors of *T*. *marneffei*. To test this hypothesis, we systematically knocked down *MP1* and its 13 homologs in *T*. *marneffei* and examined their roles in virulence in a mouse model. We demonstrated that Mp1p, but not its homologs, is a key virulence factor of *T*. *marneffei* and its virulence is achieved by improving the survival of *T*. *marneffei* in macrophages.

## Methods

### Ethics statement

The experimental protocols were approved by the Committee on the Use of Live Animals in Teaching and Research, The University of Hong Kong, in accordance with the Guidelines laid down by the NIH in the USA regarding the care and use of animals for experimental procedures.

### Strains

All fungal strains are summarized in Table 1. *T*. *marneffei* PM1 and the genetically- modified derivatives of PM1 were grown on Sabouraud dextrose agar (SDA) (Oxoid), while *Pichia pastoris* GS115 and its derivatives were grown on yeast extract peptone dextrose agar (Sigma).

**Table 1:** Fungal strains used in this study

Strains	Abbreviation	Characteristics	Relative gene expression level (%)	Source or reference
*Talaromyces marneffei*				
PM1		Human strain isolated from an HIV-negative patient		[18]
*MP1* knockdown mutant	shRNA *MP1*	PM1 derivative with *MP1* knockdown	45.3±11.4	This study
*MPLP1* knockdown mutant	shRNA *MPLP1*	PM1 derivative with *MPLP1* knockdown	4.7±3.6	This study
*MPLP2* knockdown mutant	shRNA *MPLP2*	PM1 derivative with *MPLP2* knockdown	21.6±5.6	This study
*MPLP3* knockdown mutant	shRNA *MPLP3*	PM1 derivative with *MPLP3* knockdown	32.7±6.9	This study
*MPLP4* knockdown mutant	shRNA *MPLP4*	PM1 derivative with *MPLP4* knockdown	57.6±2.8	This study
*MPLP5* knockdown mutant	shRNA *MPLP5*	PM1 derivative with *MPLP5* knockdown	8.9±5.5	This study
*MPLP6* knockdown mutant	shRNA *MPLP6*	PM1 derivative with *MPLP6* knockdown	4.7±0.9	This study
*MPLP7* knockdown mutant	shRNA *MPLP7*	PM1 derivative with *MPLP7* knockdown	23.3±9.5	This study
*MPLP8* knockdown mutant	shRNA *MPLP8*	PM1 derivative with *MPLP8* knockdown	48.4±1.7	This study
*MPLP9* knockdown mutant	shRNA *MPLP9*	PM1 derivative with *MPLP9* knockdown	19.3±5.2	This study
*MPLP10* knockdown mutant	shRNA *MPLP10*	PM1 derivative with *MPLP10* knockdown	22.6±7.9	This study
*MPLP11* knockdown mutant	shRNA *MPLP11*	PM1 derivative with *MPLP11* knockdown	34.1±9.0	This study
*MPLP12* knockdown mutant	shRNA *MPLP12*	PM1 derivative with *MPLP12* knockdown	23.9±12.7	This study
*MPLP13* knockdown mutant	shRNA *MPLP13*	PM1 derivative with *MPLP13* knockdown	57±8.3	This study
*MP1* knockout mutant	△*MP1*	PM1 derivative with *MP1* knockout	45.3±11.4	This study
*MP1* complemented mutant	△*MP1*(pAN8-1 *MP1*)	*MP1* knockout mutant derivative with *MP1* complemented		This study
*Pichia pastoris*				
GS115		*Pichia pastoris* strain for protein expression		Purchased from Invitrogen
GS115-*MP1*		GS115 derivative with *Mp1p* expression		This study

### Identification of Mp1p homologs in *T*. *marneffei*

Mp1p homologs in the *T*. *marneffei* genome were identified using TBLASTN searches with Mp1p as query [21]. Phylogenetic relationships of Mp1p homologs [MpLp1 (Mp1p-Like protein 1) to MpLp13] and Mp1p were determined using maximum likelihood method with Mega 5 [22].

### Knockdown of Mp1p homologs in *T*. *marneffei*

DNA extraction and plasmid construction were performed as previously described [23–25]. Expression vector pSilent-1, which can express the short hairpin RNAs (shRNA) against target gene, was used to construct pKD-*MP1* and pKD-*MPLP1* to *13* for *MP1* homolog knockdown. Firstly, the internal gene fragments (sense) were amplified using primers LPW9895, LPW9896, LPW11195, LPW11196, LPW11199, LPW11200, LPW11203, LPW11204, LPW11207, LPW11208, LPW11211, LPW11212, LPW11215, LPW11216, LPW11219, LPW11220, LPW11223, LPW11224, LPW11227, LPW11228, LPW11231, LPW11232, LPW11235, LPW11236, LPW11239, LPW11240, LPW11243 and LPW11244 (S1 Table) (Invitrogen). The PCR mixture (25 μl) contained *T*. *marneffei* DNA, PCR buffer (10 mM Tris-HCl pH 8.3, 50 mM KCl, 2 mM MgCl_2_ and 0.01% gelatin), 200 μM of each dNTPs and 1.0 U *Taq* polymerase (Applied Biosystem). The mixtures were amplified in 32 cycles of 95°C for 30 seconds, 56°C for 30 seconds and 72°C for 40 seconds, and a final extension at 72°C for 10 minutes (Applied Biosystem). The PCR products were purified using the QIAquick Gel Extraction kit (Qiagen), digested with *Xho*I and *Hin*dIII, and cloned into the *Xho*I-*Hin*dIII site of the pSilent-1 plasmid, resulting in pKD-*MP1*-1 and pKD-*MPLP1*-1 to pKD-*MPLP13*-1. Second, the internal gene fragments (antisense) were amplified with primers LPW 9897, LPW10358, LPW11197, LPW11198, LPW11201, LPW11202, LPW11205, LPW11206, LPW11209, LPW11210, LPW11213, LPW11214, LPW11217, LPW11218, LPW11221, LPW11222, LPW11225, LPW11226, LPW11229, LPW11230, LPW11233, LPW11234, LPW11237, LPW11238, LPW11241, LPW11242, LPW11245 and LPW11246 (S1 Table), using the PCR conditions described above. Amplified fragments were purified as described above, digested with *Bgl*II and *Kpn*I, and cloned into the *Bgl*II-*Kpn*I sites of pKD-*MP1*-1 and pKD-*MPLP1*-1 to pKD-*MPLP13*-1 respectively, resulting in pKD-*MP1* and pKD-*MPLP1* to *13*.

pKD-*MP1* and pKD-*MPLP1* to *13* were linearized using *Eco*ICRI and transformed into PM1 respectively. Transformation of *T*. *marneffei* was achieved by heat shock using the yeast form of *T*. *marneffei*. *T*. *marneffei* yeast cells obtained from cultures grown on SDA at 37°C for 10 days were used to inoculate 50 ml yeast extract peptone dextrose (YPD) broth in a 250 ml conical flask with shaking in a gyratory shaker and were further incubated at 37°C with shaking at 200 rpm for 24 hours. *T*. *marneffei* yeast cells were harvested by centrifugation at 2,500 rpm for 5 minutes at 4°C and then washed with TE buffer (10mM Tris-HCl pH7.5, 1mM EDTA) and Li-TE buffer (0.1 M lithium acetate in TE pH7.5). *T*. *marneffei* yeast cells were resuspended in 200 μl Li-TE buffer and 50 μl of yeast cells were used in each reaction. Three hundred microliters of 40 weight/volume percent (w/v %) freshly prepared polyethylene glycol (PEG) 4000 (Sigma), 5 μl of 10 mg/ml single-stranded sheared salmon sperm DNA (Invitrogen), and 1–2 μg linearized plasmid were sequentially added and mixed with *T*. *marneffei* yeast cells and the reactions were subsequently incubated at 30°C for 30 minutes and then at 42°C for 40 minutes. After the heat shock process, yeast cells were collected by three short spins at room temperature and the yeast pellets were resuspended in 10 ml of YPD broth and incubated at 37°C with shaking at 200 rpm for 24 hours. *T*. *marneffei* transformants were plated onto SDA containing 150 μg/ml hygromycin B (Invitrogen) and incubated at 37°C for 10–14 days for selecting the knockdown strains *MP1* knockdown mutant and *MPLP1-13* knockdown mutant. The RNA of the respective transformants was extracted, reverse transcribed, and checked by real-time quantitative RT-PCR (qRT-PCR). The relative gene expression levels of each knockdown mutant compared to PM1 were calculated using 2^-ΔΔCT^ method [26].

### Generation of *MP1* knockout mutant

For deletion of *MP1*, pKO-*MP1* was generated using a homologous recombination method as previously described [27]. Two DNA fragments, comprising the 1313-bp upstream and the 1406-bp downstream flanking sequences of *MP1*, were generated by PCR using LPW2558/2559 and LPW2560/2561 respectively (S1 Table). The PCR products of upstream/downstream flanking fragments were ligated into *Bgl*II/*Hin*dIII sites of vector pAN7-1 that harbored the hygromycin B resistance gene to generate plasmid pKO-*MP1*, which was then linearized with *Asp*EI and used for transformation. Hygromycin-resistant colonies were screened for homologous recombination by amplification of two fragments which harbored partial genomic sequence, *MP1* upstream/downstream fragment and vector sequence using primers LPW2815/LPW2575 and LPW392/LPW2816, whereas one set of gene-specific primers (LPW2562/LPW2772) was used to confirm successful target gene knockout (S1 Table).

### Mp1p expression by western blot analysis

Western blot analysis was performed as previously described [15]. Twenty micrograms of protein from the cell lysates of *T*. *marneffei* was loaded onto a sodium dodecyl sulfate–10% polyacrylamide gel and the proteins were subsequently onto a nitrocellulose membrane (Bio-Rad). The blot was incubated with 1:1000 dilution of guinea pig anti-Mp1p antibodies, followed by 1:4000 dilution of goat anti-guinea pig IgG (H+L) secondary antibody conjugated with horse radish peroxidase (HRP). Antigen-antibody interaction was then detected with an enhanced chemiluminescence fluorescence system (GE healthcare).

### Mp1p expression by ELISA

ELISA was performed as previously described [28]. Briefly, microwell plates (Corning) were coated with 100 μl/well of Mp1p monoclonal antibodies by incubation overnight at 4°C followed by incubation with a blocking reagent containing 2.5 g casein sodium salt, 1.21 g Tris-base, 2 g gelatin, 20 g sucrose, 0.2 g merthiolate, and 5 ml Tween 20 in 1000 ml dH_2_O (Sigma). The blocking solution was then removed and 100 μl of culture filtrates of wild type or mutant *T*. *marneffei* was serially diluted in 1:10 in 0.1% bovine serum albumin and incubated at 37°C for 1 hour. After the plates were washed, biotinylated monoclonal antibody (100 μl/well) was added and the plates were incubated for 30 minutes at 26°C. Following incubation with streptavidin-HRP (Sigma), 3,3′,5,5′-tetramethylbenzidine substrate was added. The reaction was stopped after 10 minutes by addition of 0.3 N sulfuric acid, and the plates were examined in an ELISA plate reader (Bio-Tek) at 450 nm.

### Southern blot analysis of homologous recombination

Southern blot analysis was performed as previously described [29]. For *MP1*-knockout mutant, homologous recombination at the desired locus was confirmed by Southern blot analysis of *Spe*I-digested genomic DNA probed with a 625-bp PCR product, generated by primers LPW5140/5141 (S1 Table), located at the 5’ upstream flanking region of *MP1*. Deletion of *MP1* was further confirmed by Southern blot analysis with a 680-bp PCR product, generated by primers LPW5142/2772 (S1 Table), which targeted nucleotides 191–650 of the *MP1* gene.

### Complementation of Mp1p in *MP1* knockout mutant

To examine whether the virulence properties of Mp1p can be restored in *MP1* knockout mutant, the *MP1* gene was complemented in the *MP1* knockout mutant. Plasmid pAN8-1 was used to construct pAN8-1*MP1* for *MP1* complementation. The promoter region of *A*. *nidulans gpd* gene and the terminator region of the *A*. *nidulans trpC* gene were ligated to the 5’ and 3’ ends of *MP1* gene respectively. The *MP1* fragment containing promoter and terminator was cloned into *Nar*I and *Nde*I sites of vector pAN8-1 that harbored the *Streptococcus hindustanus* phleomycin resistance gene using primers LPW19020/ LPW18915 to give plasmid pAN8-1*MP1* (S1 Table). The pAN8-1-*MP1* was linearized with *Pci*I and used for transformation.

*T*. *marneffei* strain *MP1* knockout mutant was transformed with linearized pAN8-1*MP1*, using 100 μg/ml phleomycin (Invivogen) for selection, generating *MP1* complemented mutant. Successful complementation of *MP1* gene and Mp1p production were confirmed by PCR, Western blot and ELISA respectively.

### Construction of *P*. *pastoris* expressing Mp1p

*P*. *pastoris* GS115 expressing Mp1p was generated using the Multi-Copy Pichia Expression Kit (Invitrogen). The coding region of *MP1* was amplified using primers in S1 Table, digested with *Eco*RI and *Xho*I and cloned into the *Eco*RI-*Xho*I sites of pPIC9K (Invitrogen) to generate pPIC9K-*MP1*. The plasmid was first transformed and propagated in *Escherichia coli* BL21(DE3), followed by transformation into GS115 to generate GS115-*MP1*. Mp1p expression was induced with buffered methanol complex medium at 30°C with shaking at 300 rpm for 24 hours and expression was confirmed by western blot.

### Relative gene expression by real-time quantitative RT-PCR (qRT-PCR)

Total RNA was extracted using RiboPure-Yeast (Ambion). Extracted RNA was eluted in 70 μl of RNase-free water and then used as the template for real-time qRT-PCR. Reverse transcription was performed using the SuperScript III kit (Invitrogen). Real-time qRT-PCR was performed as described previously [30], with primers as listed in S1 Table and using actin primers LPW20631/LPW20160 for normalization. cDNA was amplified in a LightCycler 2.0 (Roche) with 20 μl reaction mixtures containing FastStart DNA Master SYBR Green I Mix reagent kit (Roche), 2 μl cDNA, 2 mM MgCl_2_ and 0.5 mM primers at 95°C for 10 minutes followed by 50 cycles of 95°C for 10 seconds, 57°C (55°C for actin gene) for 5 seconds and 72°C for 23 seconds (36 seconds for actin gene). All experiments were performed in triplicates.

### Animal experiments

Balb/c (H-2^d^) mice (6-8-week-old, 18–22 g) were housed under standard conditions as described previously [23, 31]. Ten mice each were challenged intravenously with 8×10^6^ spores of PM1, *MP1* knockout mutant, *MP1* complemented mutant, *MP1* knockdown mutant and the *MPLP1-13* knockdown mutants; and 1×10^7^ spores of GS115 and GS115-*MP1* according to viable counts. Mice survival was recorded daily for 60 days. All experiments were performed in triplicates.

Five mice from the four groups challenged with PM1, *MP1* knockout mutant, *MP1* complemented mutant and *MP1* knockdown mutant were sacrificed on day 12 post-challenge. Five mice from the two groups challenged with GS115 and GS115-*MP1* were sacrificed at 24 hours post-challenge. One half of each organ was homogenized in 1× PBS for colony counts, and the other half fixed in 10% neutral buffered formalin and embedded in paraffin. Paraffin-embedded sections were stained with hematoxylin & eosin (H&E), Grocott’s methenamine silver (GMS) or Periodic acid-Schiff (PAS).

### Intracellular survival of *T*. *marneffei*

Murine macrophage-like cell line J774 (ATCC no. TIB-67) was maintained in Dulbecco's Modified Eagle's Medium (DMEM) (Gibco) supplemented with 10% heat-inactivated fetal bovine serum (Gibco) in 5% CO_2_ at 37°C in 75 cm^2^ tissue culture flask (Cellstar). J774 macrophages were differentiated by treatment with 0.32 μM phorbol myristate acetate (PMA) for 72 hours prior to the antifungal assay [23]. PMA-differentiated J774 cells were seeded in duplicates in a 24-well plate at 1×10^5^ cells/well in complete medium. Spores of *T*. *marneffei* strains PM1, *MP1* knockout mutant, *MP1* complemented mutant and *MP1* knockdown mutant were harvested and inoculated into J774 cells at 2×10^5^ spores/well (multiplicity of infection of 2) and incubated at 37°C in 5% CO_2_ incubator for 2 hours for phagocytosis. After phagocytosis, cell monolayers were washed sequentially with 240 U/ml nystatin (Sigma) and warm PBS to remove non-phagocytized Spores and maintained in DMEM supplemented with 1 μg/ml of lipopolysaccharides from *E*. *coli* serotype O111:B4 (Sigma) and 400 U/ml of recombinant mouse interferon-γ (R&D System) and further incubated for 48 hours. J774 cells were then harvested and lysed with 1% (w/v) Triton X-100 (Sigma) for colony forming unit (CFU) count at 2 hours, 8 hours, 16 hours, 24 hours and 48 hours post-inoculation. Macrophages lysed with 1% Triton X100 which consisted of the phagocytized yeasts were plated in serial dilutions in duplicate in SDA and incubated for 5 days at 37°C. The results were expressed as mean CFU ± standard deviations from three different experiments.

### Statistical analyses

Means between groups were compared with Student’s t-test. Survival of mice was tested by Kaplan-Meier method and Log-rank test.

## Results

### *T*. *marneffei* possesses 13 Mp1p homologs

**Fig 1:**
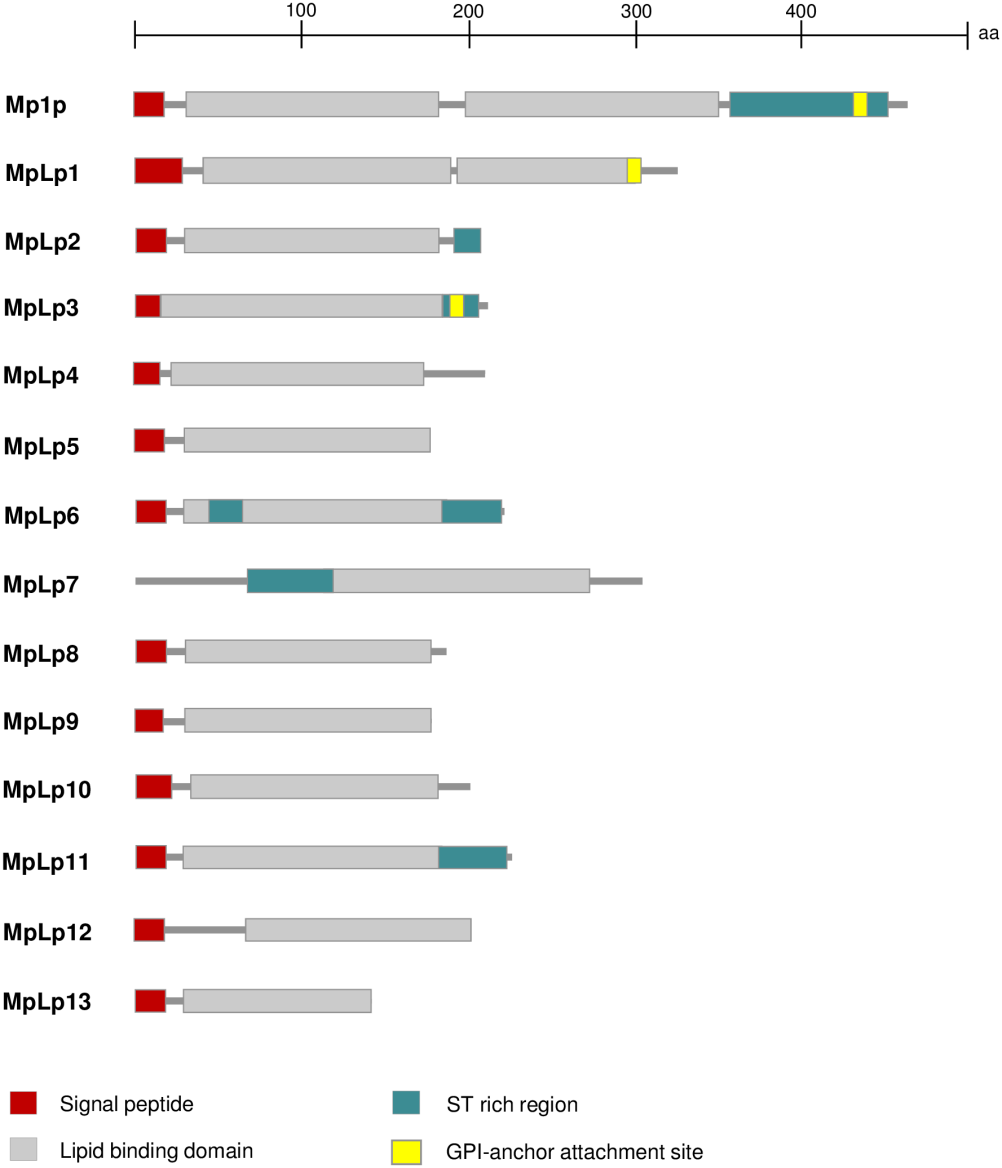
Predicted domains of Mp1p homologs in *T*. *marneffei*.

**Fig 2:**
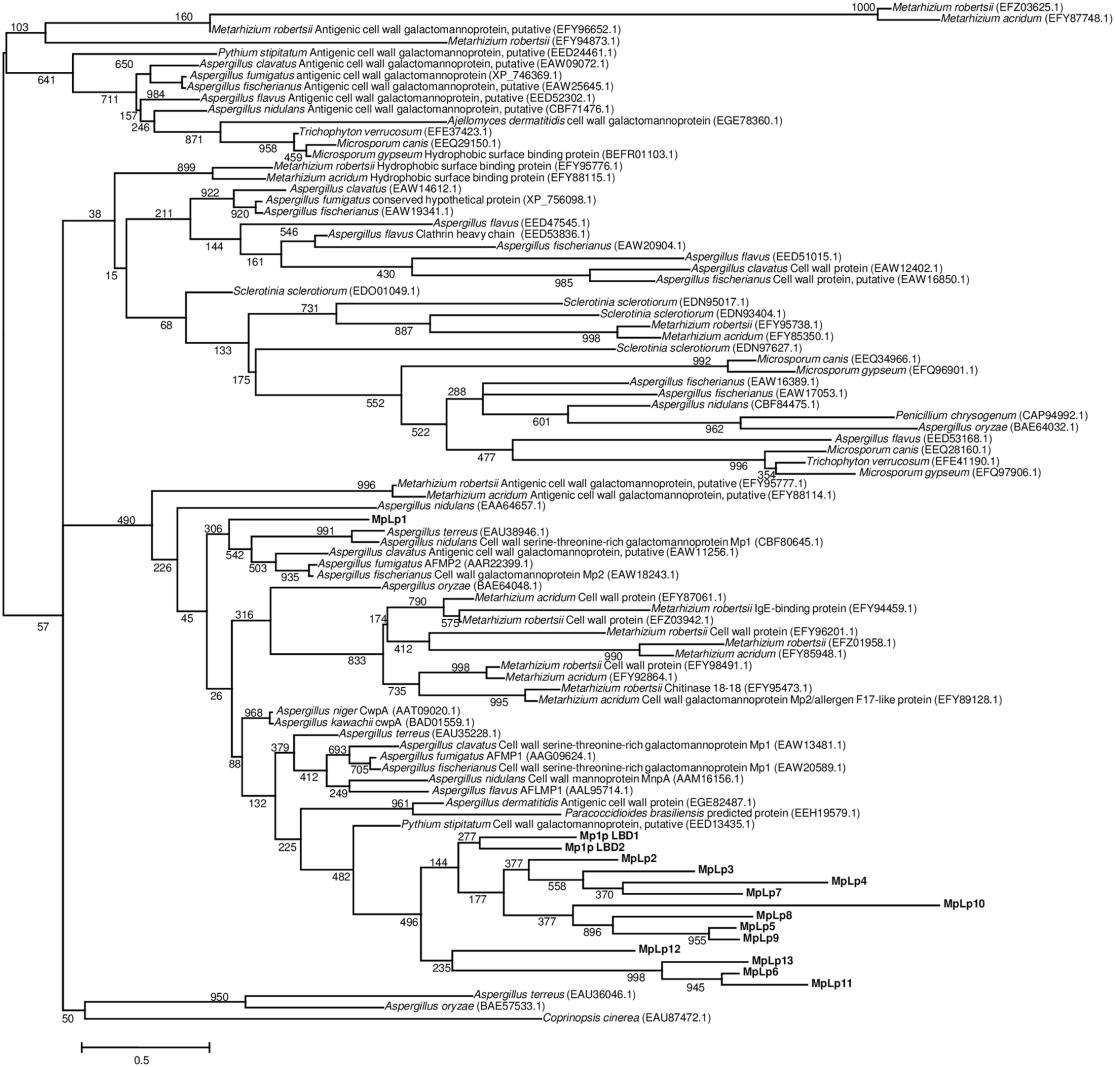
Phylogenetic analysis of the putative LBD of Mp1p homologs in *T*. *marneffei* and orthologs in other fungi. Homologs of Mp1p in *T*. *marneffei* are shown in bold. The tree was constructed by maximum likelihood method with bootstrap values calculated from 1,000 trees and rooted on midpoint. The scale bar indicates the branch lengths that correspond to 0.5 substitutions per site as indicated. Names and accession numbers are given as cited in the GenBank database.

Using TBLASTN searches and the amino acid sequence of Mp1p as query, we observed 13 additional open reading frames in the *T*. *marneffei* (strain PM1) draft genome (AGCC00000000) [18] that encodes for putative homologs of Mp1p [MpLp1 to MpLp13 (Table 2 and Fig 1)]. Unlike Mp1p which possesses two LBDs (Mp1p-LBD1 and Mp1p-LBD2), MpLp1 to MpLp13 have only one LBD each. Phylogenetically, Mp1p-LBD1 and Mp1p-LBD2 were clustered with high bootstrap support (Fig 2), suggesting that they are results of duplication of the Mp1p-LBD ancestor during its evolution in *T*. *marneffei*. Similar to Mp1p, most of these Mp1p homologs also contain putative signal peptides, variable numbers of putative N-glycosylation and O-glycosylation sites and glycosylphosphatidylinositol (GPI) anchors, and they are all expressed in both the yeast and mold phases of *T*. *marneffei* (S1 Fig).

**Table 2:** Characteristics of Mp1p homologs in *T*. *marneffei*.

Mp1p homolog	pI	Size (aa)	Molecular mass (kDa)	Intron	Subcellular localization	Lipid binding domain (aa)	N-glycosylation site	ST rich region	Signal peptide	GPI-anchor
Mp1p	5.38	462	47.8	0	Extracellular	151	1	354–447	Yes	Yes
MpLp1	4.73	324	31.3	1	Extracellular	148	1	193–299	Yes	Yes
MpLp2	4.79	206	22.5	0	Extracellular	152	0	188–203	Yes	No
MpLp3	5.16	210	23.4	0	Extracellular	168	2	187–208	Yes	Yes
MpLp4	5.33	205	19.6	0	Cytoplasmic	151	0	No	No	No
MpLp5	5.94	176	19.4	0	Extracellular	147	0	No	Yes	No
MpLp6	6.51	220	24.1	0	Extracellular	157	1	44–63; 183–218	Yes	No
MpLp7	8.46	303	33.9	1	Nuclear	159	1	68–118	No	No
MpLp8	8.71	186	20.7	0	Extracellular	147	1	No	Yes	No
MpLp9	8.89	176	19.6	0	Extracellular	147	0	No	Yes	No
MpLp10	8.93	200	22.3	1	Extracellular	148	1	No	Yes	No
MpLp11	8.98	224	24.1	0	Extracellular	155	1	182–222	Yes	No
MpLp12	9.32	201	22.2	0	Extracellular	128	0	No	Yes	No
MpLp13	5.57	218	23.3	0	Extracellular	153	0	No	Yes	No

### Mp1p is a virulence factor of *T*. *marneffei*

**Fig 3:**
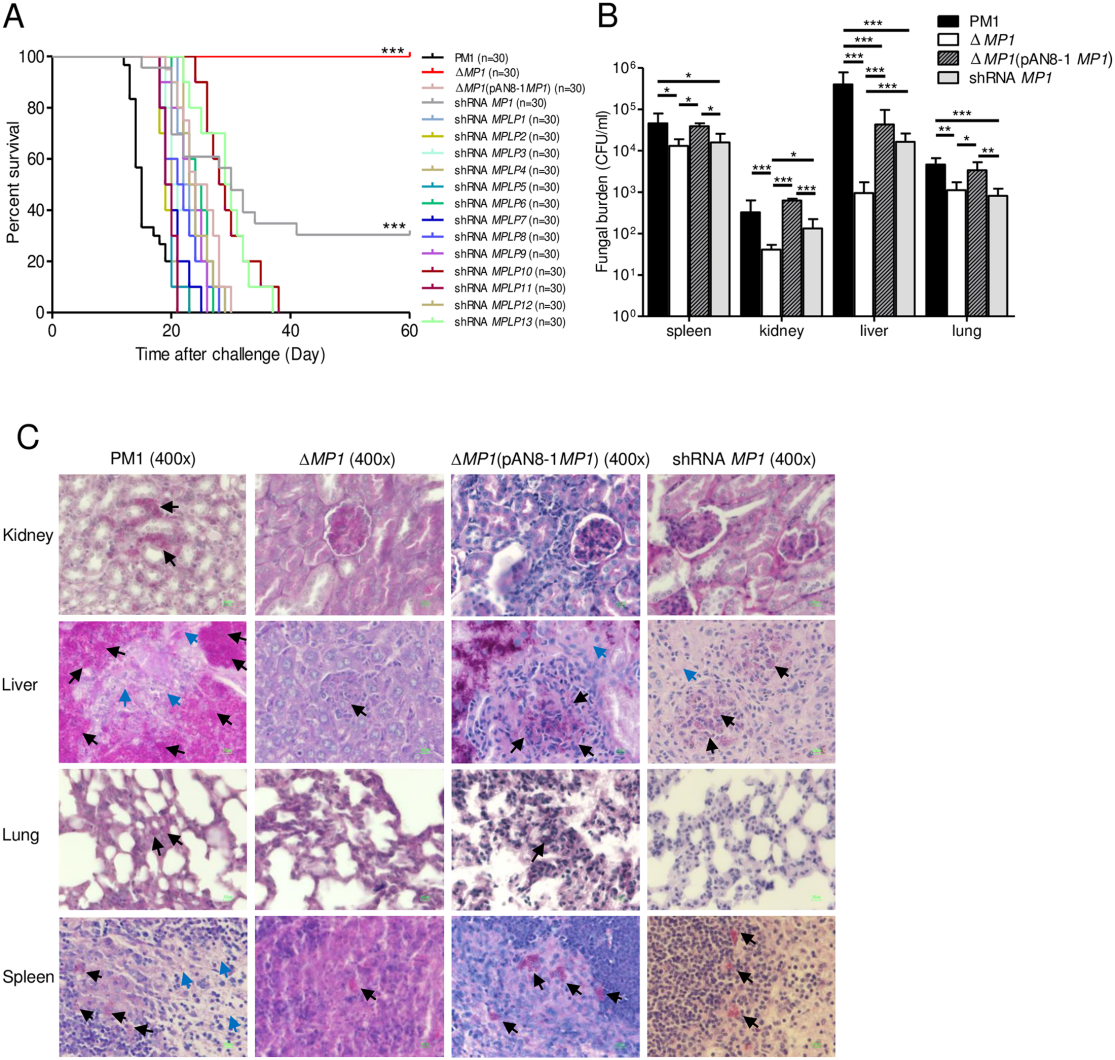
Mp1p is a virulence factor of *T*. *marneffei*. (A) Survival curves of Balb/c mice challenged with *T*. *marneffei* PM1, *MP1* knockout mutant, *MP1* complemented mutant and knockdown mutants (***P<0.0001). (B) Mean fungal burden in spleen, kidney, liver and lung of mice challenged with *T*. *marneffei* PM1, *MP1* knockout mutant, *MP1* complemented mutant and *MP1* knockdown mutant at day 12 post-challenge (***P<0.001, **P<0.01, *P<0.05). Error bars represent standard deviations. (C) Histopathological examination of PAS stained internal organs of mice at day 12 post-challenge. *T*. *marneffei* yeast cells are shown in black arrows and tissue necrosis in blue arrows.

We challenged Balb/c mice intravenously with spores of wild-type *T*. *marneffei* strain PM1, *MP1* knockout mutant, *MP1* complemented mutant, *MP1* knockdown mutant, and knockdown mutants of each of the 13 Mp1p homologs (*MPLP1* to *MPLP13* knockdown mutant), respectively. Site-specific knockout of *MP1* was confirmed by PCR, Southern blot, western blot and ELISA (S1 Table and S2–S5 Figs). Complementation of *MP1* was confirmed by PCR, western blot and ELISA (S1 Table and S3–S5 Figs). Knockdown of *MP1* and its homologs *MPLP1* to *MPLP13* were confirmed by the corresponding real-time qRT-PCR. All mice died 21 and 30 days after being challenged with PM1 and *MP1* complemented mutant respectively (Fig 3A). None of the mice died 60 days after challenge with *MP1* knockout mutant (P<0.0001). Seventy percent of mice died 60 days after challenge with *MP1* knockdown mutant (P<0.0001), showing a dose-response effect. All mice died after challenge with *MPLP1* to *MPLP13* knockdown mutant, suggesting that only Mp1p played a significant role in virulence.

At day 12 post-challenge, five mice from each of the PM1, *MP1* knockout mutant, *MP1* complemented mutant and *MP1* knockdown mutant groups were sacrificed for fungal counts and histopathological studies. The mean fungal loads of PM1 and *MP1* complemented mutant in the liver, lung, kidney and spleen were significantly higher than those of *MP1* knockout mutant and those of PM1 in the liver, lung and spleen were significantly higher than those of *MP1* knockdown mutant (Fig 3B). In the liver, the mean fungal loads of PM1 were >10-fold higher than those of *MP1* knockdown mutant and >100-fold higher than those of *MP1* knockout mutant. Histopathological studies showed a higher abundance of yeast in the kidney, spleen, liver and lung with more marked hepatic and splenic necrosis in mice challenge with PM1 compared to *MP1* knockout mutant and *MP1* knockdown mutant (Fig 3C). It also showed an abundance of yeast in the liver and spleen of mice challenged with *MP1* complemented mutant compared to *MP1* knockout mutant (Fig 3C).

### Mp1p enhances survival of *T*. *marneffei* in macrophages

**Fig 4:**
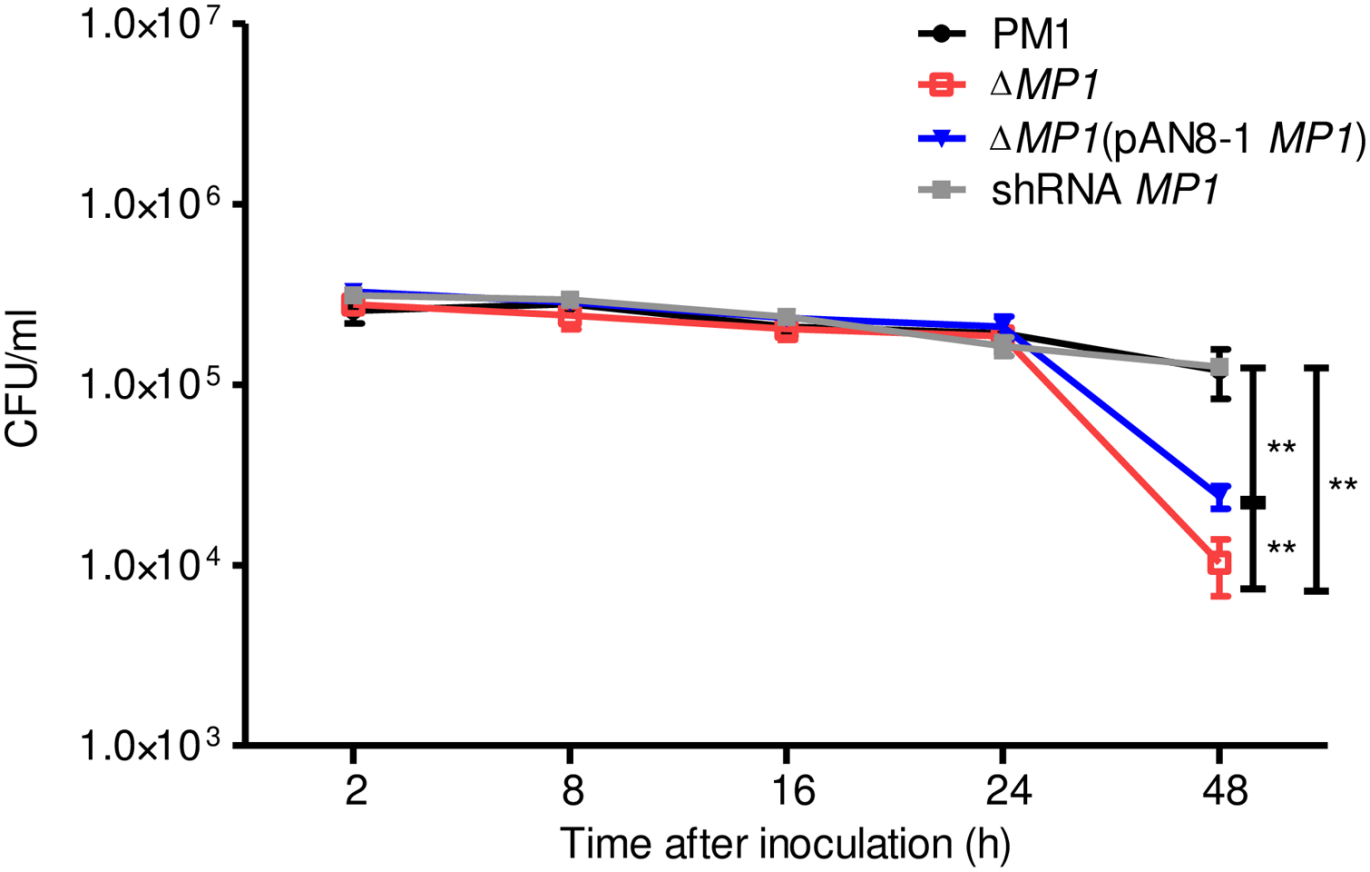
Mp1p enhances *T*. *marneffei* survival in macrophages. Intracellular survival of *T*. *marneffei* PM1, *MP1* knockout mutant, *MP1* complemented mutant and *MP1* knockdown mutant in murine macrophages at 2, 8, 16, 24 and 48 hours post-inoculation (**P<0.01). Error bars represent standard deviations.

To examine whether Mp1p can improve the intracellular survival of *T*. *marneffei* in murine macrophages, we measured the survival of PM1, *MP1* knockout mutant, *MP1* complemented mutant and *MP1* knockdown mutant in murine macrophages. PM1 and *MP1* complemented mutant survived significantly better than *MP1* knockout mutant at 48 hours (P<0.01) post-infection (Fig 4), suggesting that Mp1p mediates virulence by improving the survival of *T*. *marneffei* in macrophages, the primary defensive mechanism against the fungus.

### Mp1p improves survival of *P*. *pastoris* in mice

**Fig 5:**
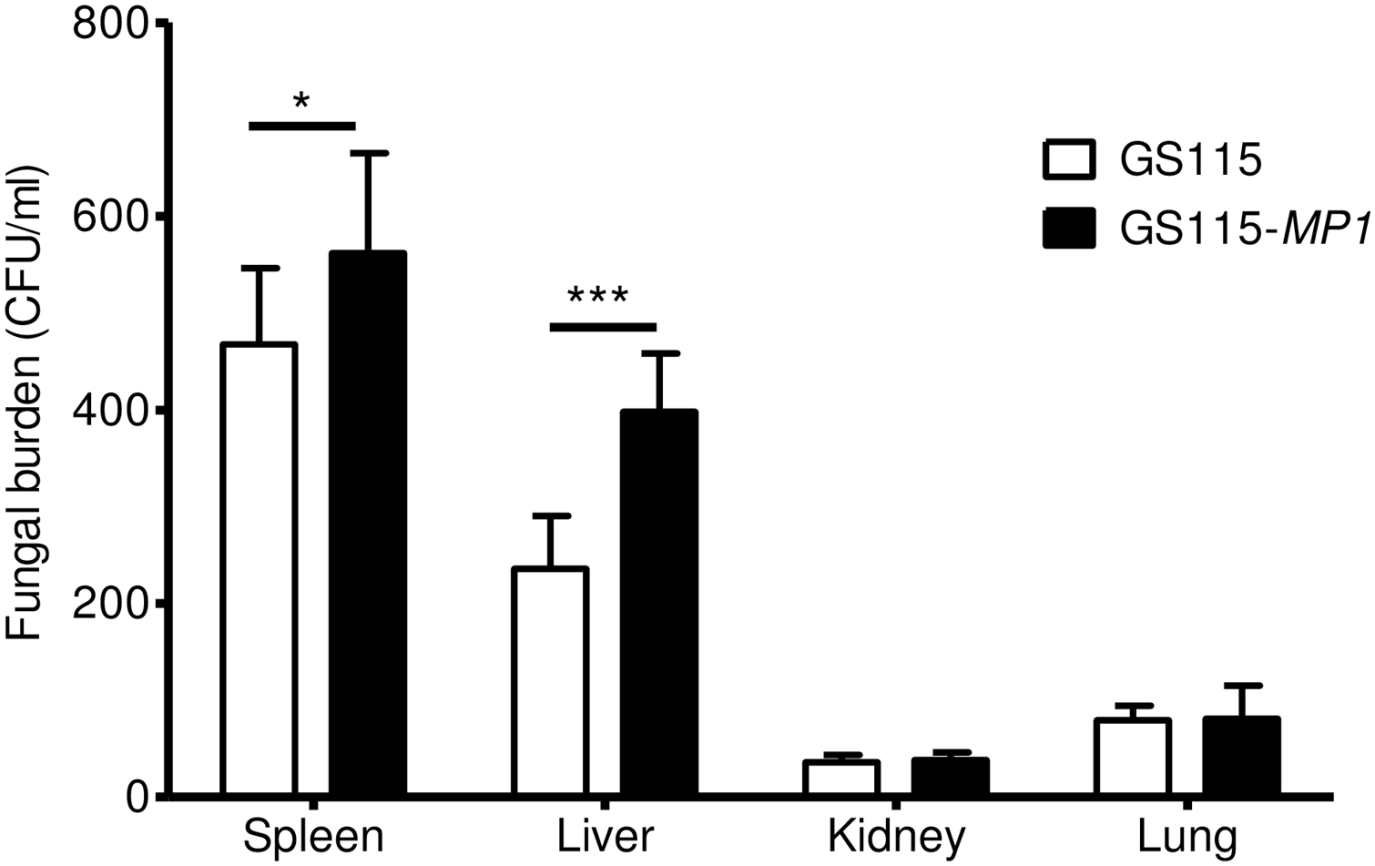
Mp1p improves survival of *Pichia pastoris* in mice. Fungal burden in spleens, livers, kidneys, and lungs of mice challenged with *P*. *pastoris* GS115 and GS115-*MP1* at 24 hours post-challenge (*P<0.05, ***P<0.001). Error bars represent standard deviations.

To determine if Mp1p can improve the survival of *P*. *pastoris* in mice, we cloned *MP1* into expression plasmid pPIC9K and transformed into *P*. *pastoris* GS115 (GS115-*MP1*) and challenged Balb/c mice with GS115 and GS115-*MP1* respectively (Table 1). The mean fungal counts of GS115-*MP1* in the liver (P<0.001) and spleen (P<0.05) of mice were significantly higher than those of GS115 at 24 hours post-challenge, indicating a gain-of-function (Fig 5).

## Discussion

In this study, we documented that Mp1p is a novel and key virulence factor of *T*. *marneffei*. In the literature, six genes (*sodA*, *cpeA*, *hsp70*, *alb1*, *pks11* and *pks12*) have been suggested to encode potential virulence factors of *T*. *marneffei* (superoxide dismutase, catalase-peroxidase, heat shock protein 70 and polyketide synthases for their biosynthetic pathways) [23, 31–34]. Among these six genes, only those encoding the polyketide synthases for the biosynthesis of melanin, mitorubrinic acid and mitorubrinol, which we characterized recently, were shown to have virulence properties in an animal model. However, knocking down of the *alb1* (for melanin biosynthesis), *pks11* (for mitorubrinic acid biosynthesis) or *pks12* (for mitorubrinol biosynthesis) gene could only rescue 10–20% of the mice, suggesting that these are not major virulence factors of *T*. *marneffei* [23, 31]. As for *sodA*, *cpeA*, and *hsp70*, studies have demonstrated that the expression of their transcripts in *T*. *marneffei* was higher during macrophage infection, oxidative stress or mycelium to yeast phase transition, although these proteins have also been implicated as virulence factors in other fungi [32–34]. In the present study, a *T*. *marneffei* strain isolated from an HIV-negative patient with the typical clinical features and with genome sequence available was used [18]. Results showed that after knocking out of *MP1* in *T*. *marneffei*, all mice survived (Fig 3A). With partial knocking down of *MP1*, a significant proportion of mice survived (Fig 3A). Moreover, the virulence properties of *T*. *marneffei* were restored by complementation of the *MP1* gene in its knockout strain. The deaths of the mice were a result of invasion of *T*. *marneffei*, as demonstrated by higher fungal counts with massive necrosis in the internal organs of mice challenged with wild-type *T*. *marneffei* as compared to both the *MP1* knockout mutant and knockdown mutants (Fig 3B and 3C). Further direct evidence to show that Mp1p is a *bona fide* virulence factor was shown by the cloning of *MP1* into *P*. *pastoris* enhanced survival of the fungus in mice was observed, indicating a gain-of-function (Fig 5).

The molecular mechanism of virulence for Mp1p remains to be determined. At the cellular level, Mp1p improved the survival of *T*. *marneffei* in macrophages (Fig 4), the key defensive cells against the fungus. Although we have shown previously that Mp1p is able to bind palmitic acid [20], this does not seem to provide a direct clue to the molecular mechanism of virulence, as *in vitro* binding of a protein to other proteins, lipids or other molecules is not uncommon and may not have physiological roles. Since palmitic acid is a fatty acid, further experiments to examine the capability of Mp1p to bind other fatty acids as well as site-directed mutagenesis experiments to look for mutants that affect both the binding activities and virulence properties of *T*. *marneffei* will help shed light on the mechanism of virulence of Mp1p. It is noteworthy that the *T*. *marneffei* genome contains 13 Mp1p homologs in addition to Mp1p. Similar to Mp1p, these 13 Mp1p homologs are also expressed in significant amounts in both the mold and yeast phases of the fungus (S1 Fig). Overall, their LBDs possessed 21–40% and 25–43% amino acid identities to those of Mp1p-LBD1 and Mp1p-LBD2 respectively and most of their LBDs are comparable in size to Mp1p (Table 2). Moreover, some of these homologs possess N-glycosylation sites, ST rich regions and GPI anchor, which are regions that are also found in Mp1p (Table 2). Interestingly, in contrast to Mp1p which is a strong virulence factor of *T*. *marneffei*, the other 13 Mp1p homologs present in the *T*. *marneffei* genome do not contribute significantly to virulence as demonstrated by the mice challenge experiments using the corresponding knockdown mutants (Fig 3). Further studies are required to determine the reason for the differential virulence properties of Mp1p and its homologs.

The virulence property of Mp1p may also be present in Mp1p homologs found in other fungi. Phylogenetic analysis of the mitochondrial genomes of *T*. *marneffei* and other fungi showed that *T*. *marneffei* is closely related to the *Aspergillus* species [25], which are highly virulent molds that cause high fatalities in patients with hematological malignancies, transplant recipients, HIV positive patients and patients on corticosteroid therapy [35, 36]. We previously showed that *A*. *fumigatus* and *A*. *flavus* both possess Mp1p homologs (Afmp1p and Afmp2p in *A*. *fumigatus* and Aflmp1p in *A*. *flavus*) and these homologous proteins in *A*. *fumigatus* and *A*. *flavus* are also immunogenic proteins which can be used for serological diagnosis in the corresponding fungi [37–41]. Since the LBDs of these proteins are homologous to Mp1p (Fig 2), we speculate that they may also help the corresponding *Aspergillus* species to evade host immunity. Further experiments will reveal the virulence spectrum of Mp1p homologs in different fungal pathogens.

## Supporting Information

S1 Fig Analysis of transcript formation of the Mp1p homologs under the two culture conditions. The constitutively expressed actin was used as control.
(TIF)

S2 Fig Southern blot of genomic DNA from the wild-type *T*. *marneffei* strain PM1 and the *MP1* knockout mutant. The genomic DNA was digested with *Spe*I and probed with 1-kb *MP1* upstream region probe and *MP1* probe. Homologous recombination of the deletion construct at the *MP1* locus resulted in integration of the hygromycin resistance gene that increased the size of the hybridizing band. (a) Wild-type *T*. *marneffei* strain PM1 and *MP1* knockout mutant probed with 680-bp *MP1* probe. (b) Wild-type *T*. *marneffei* strain PM1 and *MP1* knockout mutant probed with 625-bp 1-kb *MP1* upstream region probe.
(TIF)

S3 Fig Western blot showing expression of Mp1p. Lane 1, Wild-type *T*. *marneffei* strain PM1. Lane 2, *MP1* knockout mutant. Lane 3, *MP1* complemented mutant.
(TIF)

S4 Fig PCR and agarose gel electrophoresis detecting the presence/absence of *MP1*. Lane 1, Wild-type *T*. *marneffei* strain PM1. Lane 2, *MP1* knockout mutant. Lane 3, *MP1* complemented mutant. Lane 4, *MP1* knockdown mutant.
(TIF)

S5 Fig ELISA detecting the expression of Mp1p. Diluted culture supernatants of wild-type *T*. *marneffei* strain PM1, *MP1* knockout mutant and *MP1* complemented mutant were used for Mp1p detection in ELISA. Culture medium was used as control.
(TIF)

S1 Table Primers used in this study. (DOC)
