# Supplementary figures and images for: Mp1p Is a Virulence Factor in Talaromyces (Penicillium) marneffei
Source: PLoS Negl Trop Dis. 2016 Aug 25;10(8):e0004907. doi: 10.1371/journal.pntd.0004907 (PMC4999278; doi:10.1371/journal.pntd.0004907)

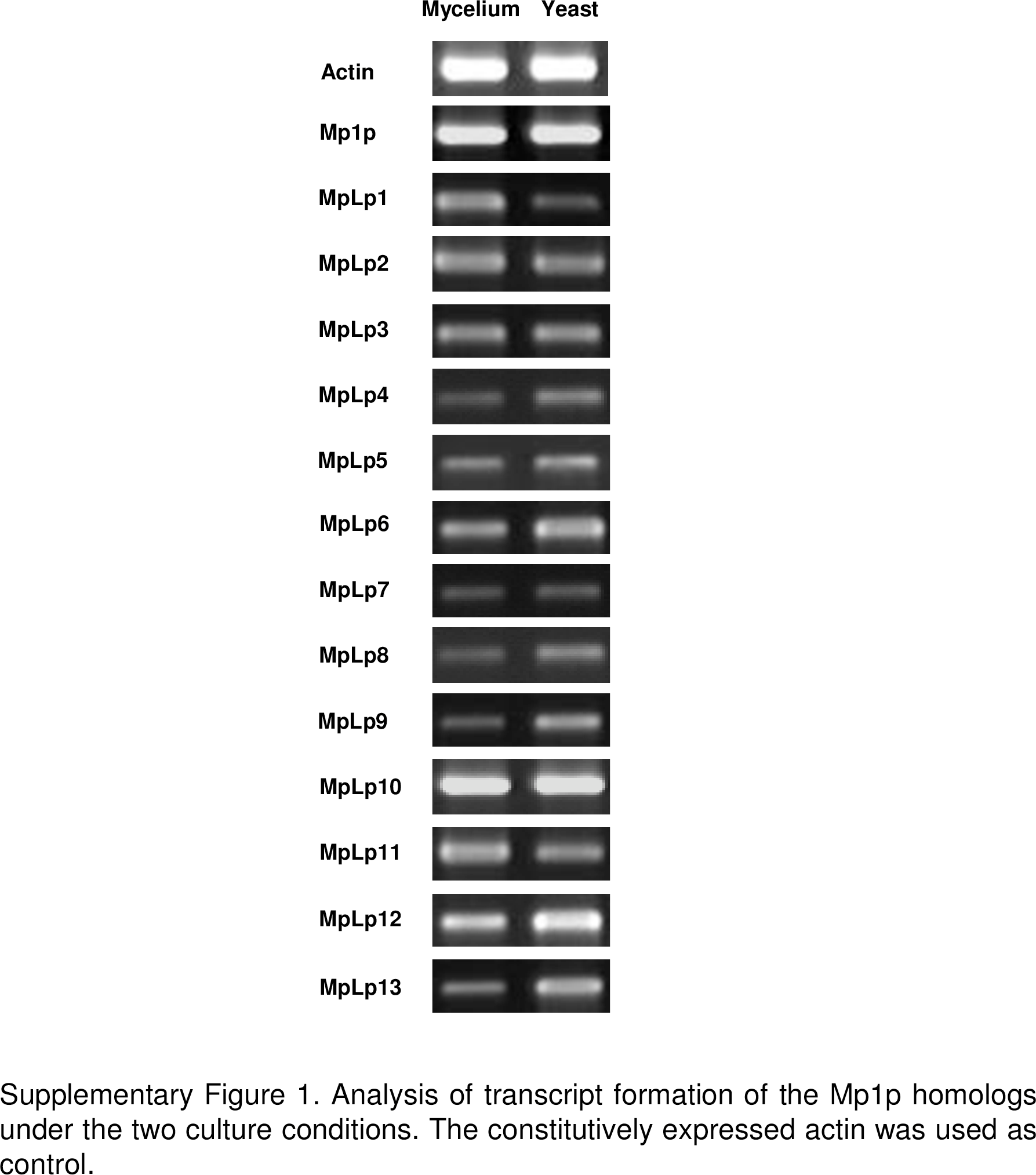

Supplement: S1 Fig — The constitutively expressed actin was used as control. (TIF) [file pntd.0004907.s001.tif]

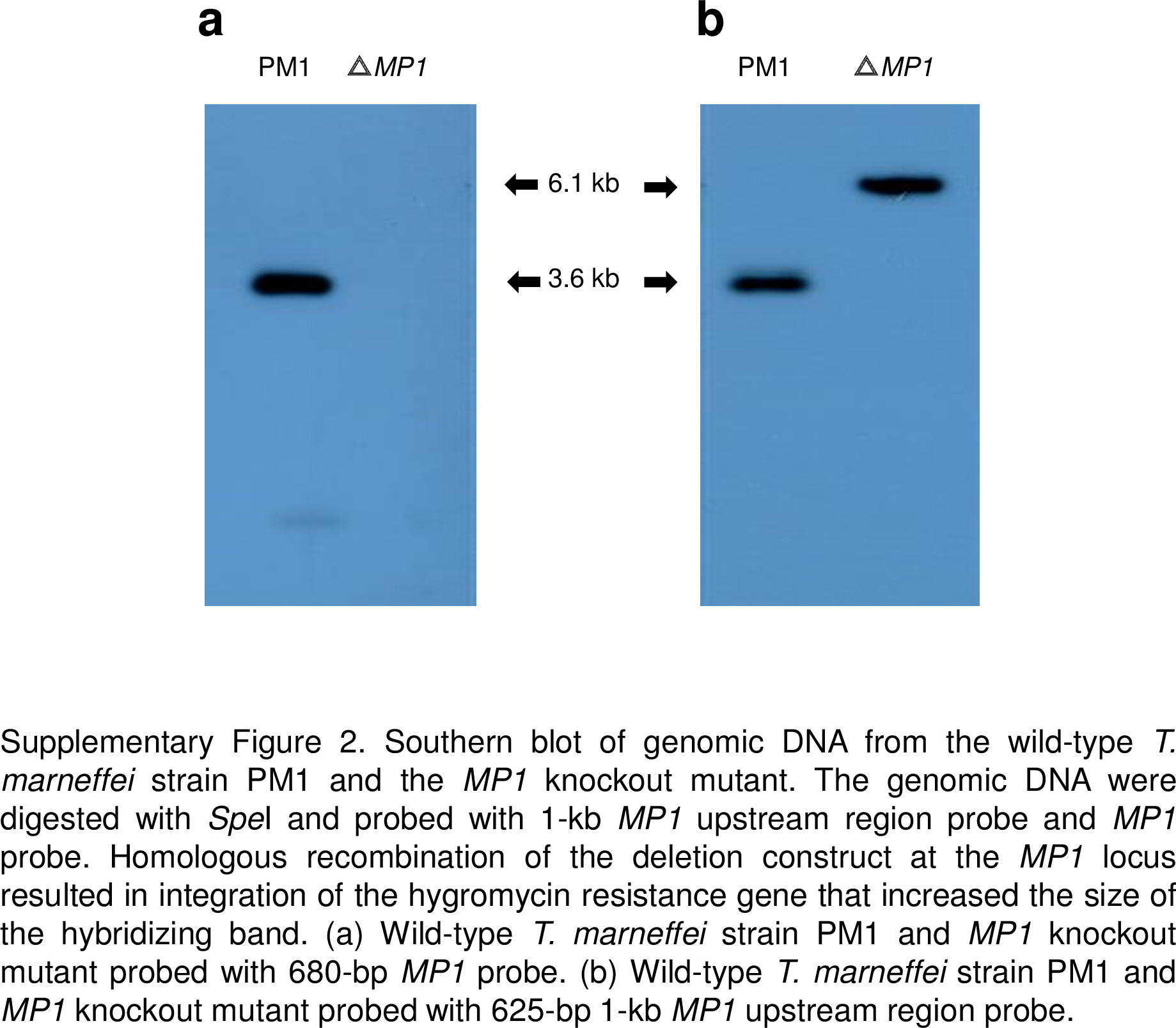

Supplement: S2 Fig — The genomic DNA was digested with SpeI and probed with 1-kb MP1 upstream region probe and MP1 probe. Homologous recombination of the deletion construct at the MP1 locus resulted in integration of the hygromycin resistance gene that increased the size of the hybridizing band. (a) Wild-type T. marneffei strain PM1 and MP1 knockout mutant probed with 680-bp MP1 probe. (b) Wild-type T. marneffei strain PM1 and MP1 knockout mutant probed with 625-bp 1-kb MP1 upstream region probe. (TIF) [file pntd.0004907.s002.tif]

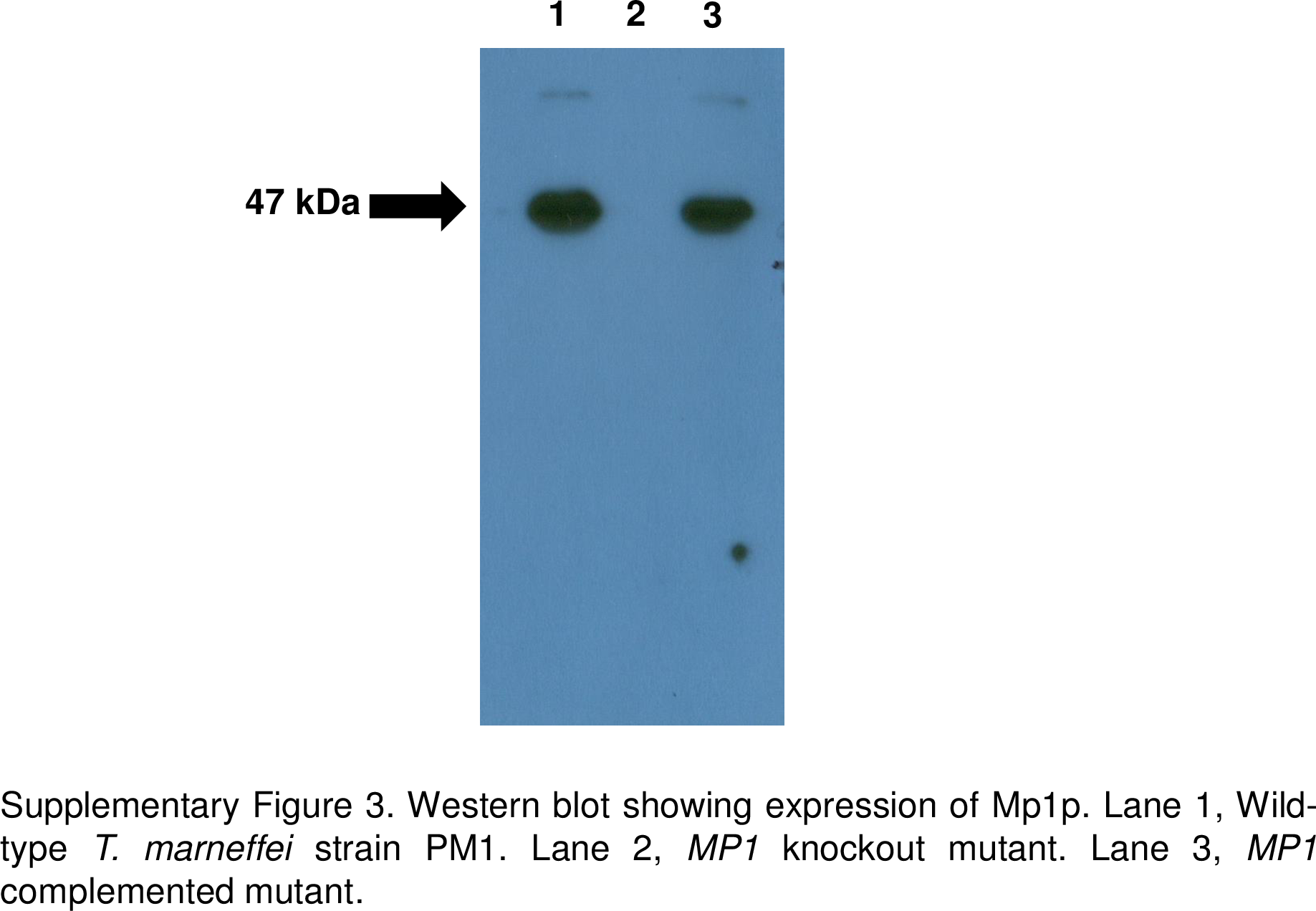

Supplement: S3 Fig — Lane 1, Wild-type T. marneffei strain PM1. Lane 2, MP1 knockout mutant. Lane 3, MP1 complemented mutant. (TIF) [file pntd.0004907.s003.tif]

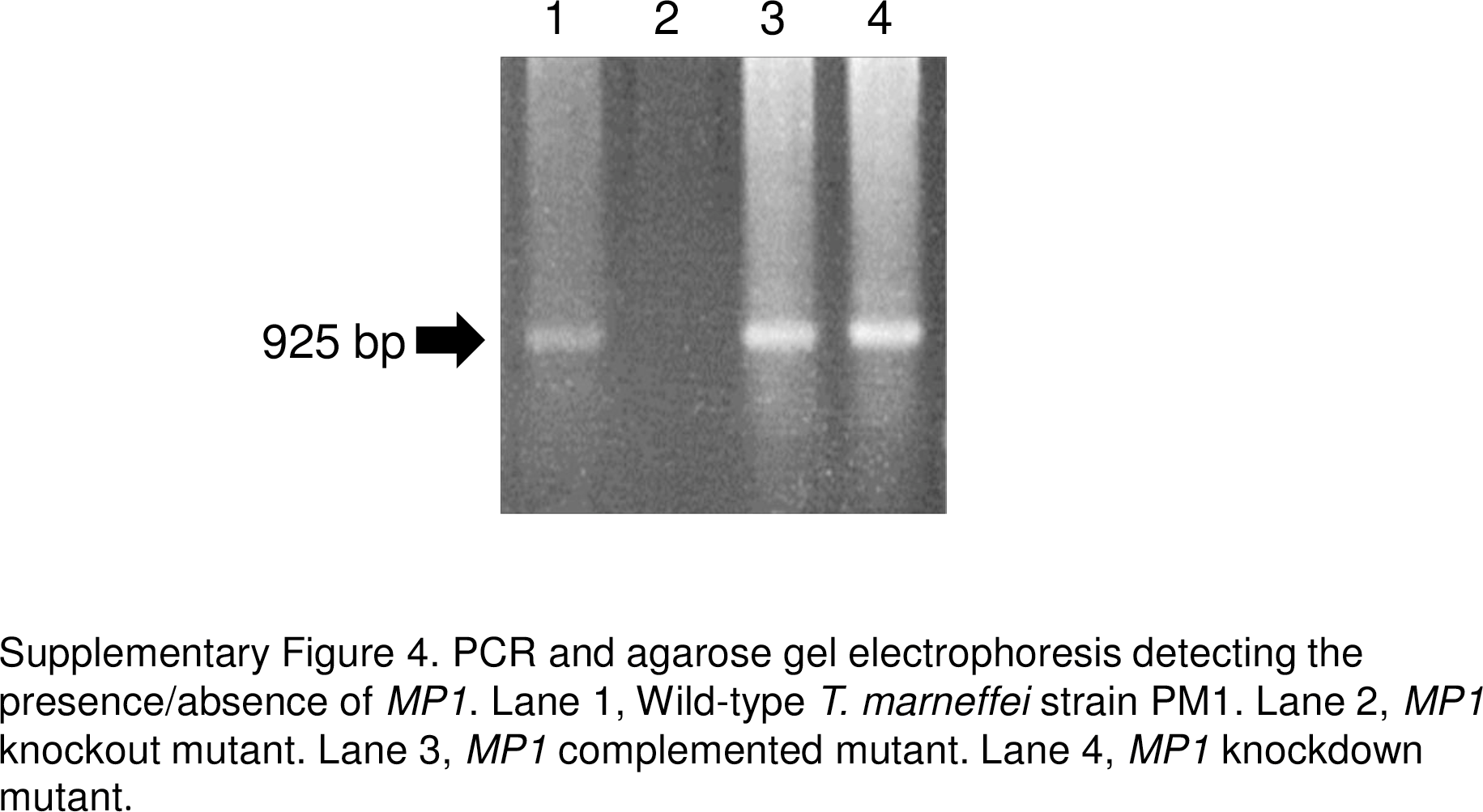

Supplement: S4 Fig — Lane 1, Wild-type T. marneffei strain PM1. Lane 2, MP1 knockout mutant. Lane 3, MP1 complemented mutant. Lane 4, MP1 knockdown mutant. (TIF) [file pntd.0004907.s004.tif]

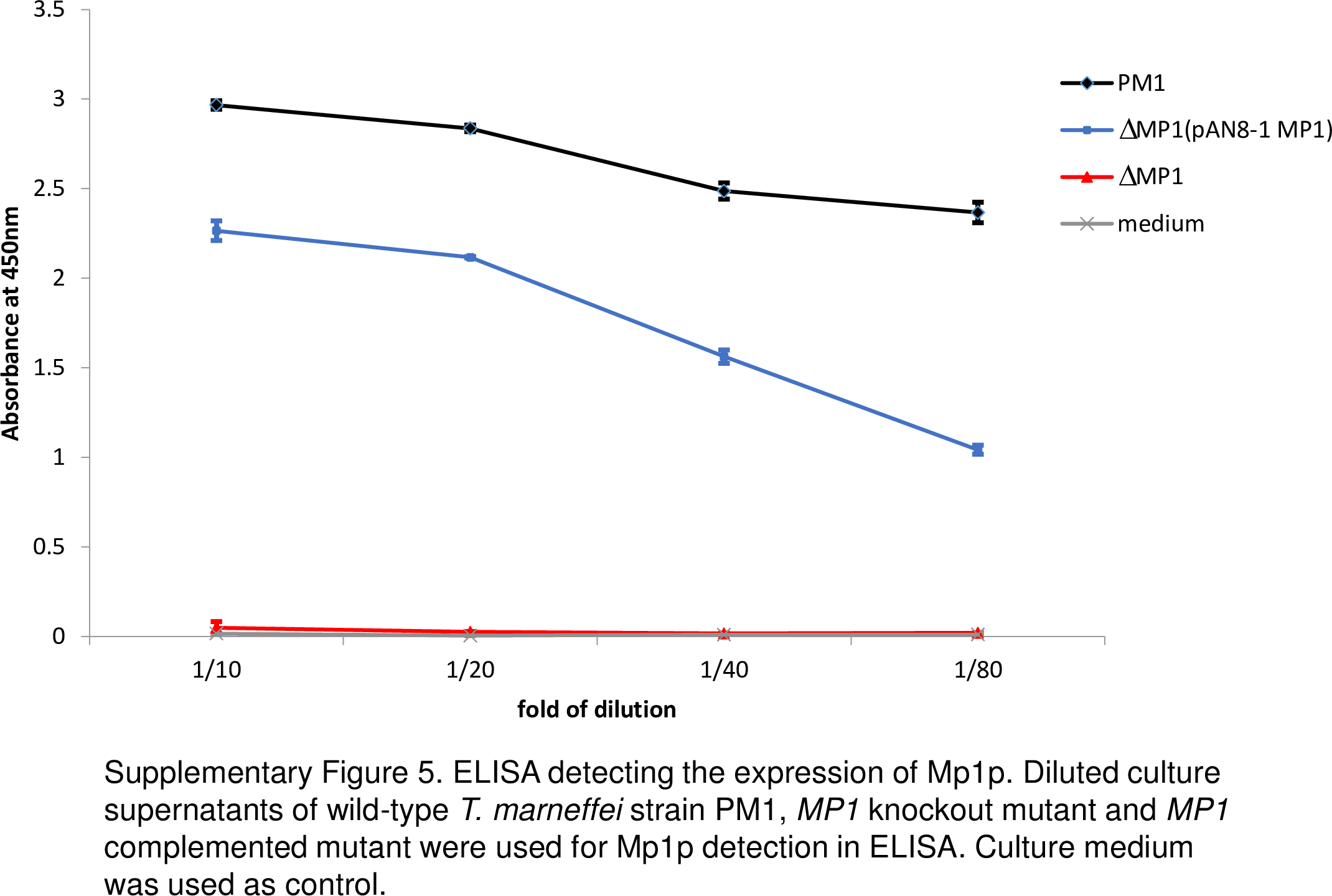

Supplement: S5 Fig — Diluted culture supernatants of wild-type T. marneffei strain PM1, MP1 knockout mutant and MP1 complemented mutant were used for Mp1p detection in ELISA. Culture medium was used as control. (TIF) [file pntd.0004907.s005.tif]
