# Supplementary material for: Mp1p Is a Virulence Factor in Talaromyces (Penicillium) marneffei
Source: PLoS Negl Trop Dis. 2016 Aug 25;10(8):e0004907. doi: 10.1371/journal.pntd.0004907 (PMC4999278; doi:10.1371/journal.pntd.0004907)
Supplement: S1 Table — (DOC) [file pntd.0004907.s006.doc]

Supplementary Table 1. Primers used in this study

| **Genes/Fragments** | **Primers** | |
| --- | --- | --- |
|  | **Forward** | **Reverse** |
| For gene knockdown of *MP1* and homologs | | |
| *MP1* *Xho*I-*Hin*dIII | LPW9896 5'-CCGCTCGAGCAATACCTTTCTTGATGCCAG-3' | LPW9895 5'-CCCAAGCTTCGTTACACTCAGCGAGTTAGTC-3' |
| *MP1* *Bgl*II-*Kpn*I | LPW10358 5'-GGGGTACCCAATACCTTTCTTGATGCCAG-3' | LPW9897 5'-GAAGATCTCGTTACACTCAGCGAGTTAGTC-3' |
| *MPLP1* *Xho*I-*Hin*dIII | LPW11195 5'-CCGCTCGAGTGTTCTTTCGGACTCAACAA-3' | LPW11196 5'-CCCAAGCTTAGTCACAGGACTAGTGGTGTCA-3' |
| *MPLP1* *Bgl*II-*Kpn*I | LPW11197 5'-GGGGTACCTGTTCTTTCGGACTCAACAA-3' | LPW11198 5'-GAAGATCTAGTCACAGGACTAGTGGTGTCA-3' |
| *MPLP2* *Xho*I-*Hin*dIII | LPW11199 5'-CCGCTCGAGGAGAAGAAGTCATCAATAGCTCTC-3' | LPW11200 5'-CCCAAGCTTGAGCCCTTACGTTGATCACT-3' |
| *MPLP2* *Bgl*II-*Kpn*I | LPW11201 5'-GGGGTACCGAGAAGAAGTCATCAATAGCTCTC-3' | LPW11202 5'-GAAGATCTGAGCCCTTACGTTGATCACT-3' |
| *MPLP3* *Xho*I-*Hin*dIII | LPW11203 5'-CCGCTCGAGAAAAGCTACCTCCAGTCGC-3' | LPW11204 5'-CCCAAGCTTATCTTTGGTCTTTCCGCC-3' |
| *MPLP3* *Bgl*II-*Kpn*I | LPW11205 5'-GGGGTACCAAAAGCTACCTCCAGTCGC-3' | LPW11206 5'-GAAGATCTATCTTTGGTCTTTCCGCC-3' |
| *MPLP4* *Xho*I-*Hin*dIII | LPW11207 5'-CCGCTCGAGGCTCTTGCGAGCTCTTACA-3' | LPW11208 5'-CCCAAGCTTGAACCTGCTGGTTGATCG-3' |
| *MPLP4* *Bgl*II-*Kpn*I | LPW11209 5'-GGGGTACCGCTCTTGCGAGCTCTTACA-3' | LPW11210 5'-GAAGATCTGAACCTGCTGGTTGATCG-3' |
| *MPLP5* *Xho*I-*Hin*dIII | LPW11211 5'-CCGCTCGAGCTCCCTCATCCTGCTCAGT-3' | LPW11212 5'-CCCAAGCTTCAGCCCCTTCCAAGAATT-3' |
| *MPLP5* *Bgl*II-*Kpn*I | LPW11213 5'-GGGGTACCCTCCCTCATCCTGCTCAGT-3' | LPW11214 5'-GAAGATCTCAGCCCCTTCCAAGAATT-3' |
| *MPLP6* *Xho*I-*Hin*dIII | LPW11215 5'-CCGCTCGAGTCAGCAGATGGCTTCTATAGAC-3' | LPW11216 5'-CCCAAGCTTCTAGTTAGCACTAGCAGCATCAG-3' |
| *MPLP6* *Bgl*II-*Kpn*I | LPW11217 5'-GGGGTACCTCAGCAGATGGCTTCTATAGAC-3' | LPW11218 5'-GAAGATCTCTAGTTAGCACTAGCAGCATCAG-3' |
| *MPLP7* *Xho*I-*Hin*dIII | LPW11219 5'-CCGCTCGAGTGCACCAGTGAAAGTAGGG-3' | LPW11220 5'-CCCAAGCTTCCAAGCTATTAAGGATGGGA-3' |
| *MPLP7* *Bgl*II-*Kpn*I | LPW11221 5'-GGGGTACCTGCACCAGTGAAAGTAGGG-3' | LPW11222 5'-GAAGATCTCCAAGCTATTAAGGATGGGA-3' |
| *MPLP8* *Xho*I-*Hin*dIII | LPW11223 5'-CCGCTCGAGATACTCCTTAGTCTTTCTGCCC-3' | LPW11224 5'-CCCAAGCTTAAGTATAGATTTAAGACCAGCAGC-3' |
| *MPLP8* *Bgl*II-*Kpn*I | LPW11225 5'-GGGGTACCATACTCCTTAGTCTTTCTGCCC-3' | LPW11226 5'-GAAGATCTAAGTATAGATTTAAGACCAGCAGC-3' |
| *MPLP9* *Xho*I-*Hin*dIII | LPW11227 5'-CCGCTCGAGAGTCTTTCTGCCCAGGCT-3' | LPW11228 5'-CCCAAGCTTTCTCAAGGGAGGACCGTT-3' |
| *MPLP9* *Bgl*II-*Kpn*I | LPW11229 5'-GGGGTACCAGTCTTTCTGCCCAGGCT-3' | LPW11230 5'-GAAGATCTTCTCAAGGGAGGACCGTT-3' |
| *MPLP10* *Xho*I-*Hin*dIII | LPW11231 5'-CCGCTCGAGGTTCCTGTAGGGCTTTATTAGAG-3' | LPW11232 5'-CCCAAGCTTAGTTCTTACAGTTCTTACTCTCCCT-3' |
| *MPLP10* *Bgl*II-*Kpn*I | LPW11233 5'-GGGGTACCGTTCCTGTAGGGCTTTATTAGAG-3' | LPW11234 5'-GAAGATCTAGTTCTTACAGTTCTTACTCTCCCT-3' |
| *MPLP11* *Xho*I-*Hin*dIII | LPW11235 5'-CCGCTCGAGGAAGCAAGAATAAGCGACTTC-3' | LPW11236 5'-CCCAAGCTTTGAAGTTCTTACCCTCTCTCATC-3' |
| *MPLP11* *Bgl*II-*Kpn*I | LPW11237 5'-GGGGTACCGAAGCAAGAATAAGCGACTTC-3' | LPW11238 5'-GAAGATCTTGAAGTTCTTACCCTCTCTCATC-3' |
| *MPLP12* *Xho*I-*Hin*dIII | LPW11239 5'-CCGCTCGAGTAATAATTACCAGGGCGGAG-3' | LPW11240 5'-CCCAAGCTTTATCTCATAGGCGAACTCTCTG-3' |
| *MPLP12* *Bgl*II-*Kpn*I | LPW11241 5'-GGGGTACCTAATAATTACCAGGGCGGAG-3' | LPW11242 5'-GAAGATCTTATCTCATAGGCGAACTCTCTG-3' |
| *MPLP13* *Xho*I-*Hin*dIII | LPW11243 5'-CCGCTCGAGACTTCATCACAGCTTTTGAGAA-3' | LPW11244 5'-CCCAAGCTTTGAAGTTCTTACCCTCCCTTAT-3' |
| *MPLP13* *Bgl*II-*Kpn*I | LPW11245 5'-GGGGTACCACTTCATCACAGCTTTTGAGAA-3' | LPW11246 5'-GAAGATCTTGAAGTTCTTACCCTCCCTTAT-3' |
|  |  |  |
| For *MP1* knockout |  |  |
| *MP1* upstream *BglII* | LPW2558 5’- GGAAGATCTGATTGAGTTGTGTGGAGAAA-3’ | LPW2559 5’- GGAAGATCTGAGAGTAGACAGGATAGG-3’ |
| *MP1* downstream *Hind*III | LPW2560 5’- CCCAAGCTTATCTGTGGAAGGTTTGCTC-3’ | LPW25615’- CCCAAGCTTCCGTCTTCTCATCCTGC-3’ |
|  |  |  |
| For *MP1* knockout confirmation |  |  |
| Region harbors *MP1* 5’upstream – pAN7-1 | LPW2815 5’-ATAACTATGGAAGATCGCAGC-3’ | LPW2575 5’- GGTAGTTGTGACAGGACGAG-3’ |
| Region harbors pAN7-1- *MP1* 3’downstream | LPW392 5’- GGTTGCCTAGTGAATGCTCC-3’ | LPW2816 5’- CGCCAGACTGCCTCCGT-3’ |
| *MP1* internal region | LPW2562 5’-CGTTAATCAACATGAAGTTC-3’ | LPW2772 5’-TGTTGCCTAAGATTGTTCTC-3’ |
|  | | |
| *Pichia* expression |  |  |
| *MP1* *Eco*RI-*Xho*I | 5'-CCGCTCGAGAAAAGAATGAGCCCTTACGTTGATCACC-3' | 5'-CCGGAATTCTTAATGATGATGATGATGATGTCAATTACCGCCAGTTGGCG-3' |
|  |  |  |
| Southern blot analysis |  |  |
| *MP1* 5’upstream region | LPW5140 5’-GCTCAAATGGTGACTGACT-3’ | LPW5141 5’-GAAAGGACCTCCACTCACTA-3’ |
| *MP1* internal region | LPW5142 5’-ATTATCAAAGCCACTGAGAA-3’ | LPW2772 5’-TGTTGCCTAAGATTGTTCTC-3’ |
|  |  |  |
| Complementation of *MP1* in Δ*MP1* | |  |
| Region harbors promotor– *MP1*–terminator *Nar*I*-Nde*I | LPW19020 5’- GGCGAATGGCGCCGAATTCCCTTGTATCTCTACACAC-3’ | LPW18915 5’-GGAATTCCATATGCCAGTGCCAAGCTCTAGAAA-3’ |
|  |  |  |
| Real-time qRT-PCR |  |  |
| *Actin* | LPW20631 5’-GAACGTGAAATCGTCCGT-3’ | LPW20160 5’-AGCAAGAATGGAACCACC-3’ |
| *MP1* | LPW24441 5’-TGTAAATGTTTTCAAGAAGGTCCTC-3’ | LPW24442 5’-CGACAGTCTTCATAATCAACTTGTT-3’ |
